# Occupational Upper-Limb Injuries: A Retrospective Review of Outcomes at a Private Hospital in Gqeberha (Formerly Port Elizabeth), South Africa

**DOI:** 10.7759/cureus.98760

**Published:** 2025-12-08

**Authors:** Javed IK Niazi, Abdirashid A Aden, Aftab Younus

**Affiliations:** 1 Orthopaedic Surgery, Netcare Greenacres Hospital, Port Elizabeth, ZAF; 2 Orthopaedic Surgery, Wits University, Johannesburg, ZAF; 3 Orthopaedics and Traumatology, Helen Joseph Hospital, Johannesburg, ZAF

**Keywords:** epidemiology, industrial workers, injury prevention, occupational injuries, rehabilitation outcomes, retrospective study, return to work, south africa, upper limb trauma, workplace safety

## Abstract

Background

Occupational upper-limb injuries represent a significant burden on healthcare systems and workforce productivity globally. While developed nations have implemented comprehensive workplace safety measures, data from low- and middle-income countries, particularly South Africa, remain limited. This study addresses this knowledge gap by examining injury patterns and outcomes in a major industrial region.

Objective

To describe the epidemiology, injury mechanisms, and return-to-work outcomes of occupational upper-limb injuries managed at a private hospital in Gqeberha (formerly Port Elizabeth) in the Eastern Cape province, South Africa, between April 1, 2013, and December 31, 2020.

Methods

A retrospective review was conducted of 334 consenting patients aged ≥18 years with occupational upper-limb injuries treated by the principal author. Data collection included demographics, industry sector, injury mechanism and type, and return-to-work outcomes. Statistical analysis employed descriptive statistics and Fisher’s exact test for categorical associations, with significance set at p < 0.05.

Results

The cohort was predominantly male (267 patients, 79.9%) and right-hand dominant (320 patients, 95.8%), with 266 patients (79.6%) under 50 years of age. Manufacturing (117 patients, 35%) and service-sector workers (107 patients, 32%) were most affected. Fractures were the most common injuries (144 patients, 43.1%), followed by tendon injuries (45 patients, 13.5%) and amputations (36 patients, 10.8%). Falls (115 patients, 34.4%) and crush injuries (114 patients, 34.1%) were the predominant mechanisms. Younger workers (18-30 years) had a significantly higher amputation rate, while workers over 50 years showed increased rates of rotator cuff tears and dislocations (p = 0.0025). Return-to-work outcomes were favorable, with 267 patients (80%) resuming original roles; however, 30 patients (9%) experienced job loss. Rotator cuff tears required the longest recovery period (mean 59.3 ± 28.7 days).

Conclusions

Occupational upper-limb injuries predominantly affected young male workers in manufacturing and service industries. The study identifies specific age-related injury patterns and temporal risk factors that can inform targeted prevention strategies. While return-to-work rates were encouraging, the socioeconomic impact of job loss emphasizes the need for comprehensive workplace safety interventions and injury-specific rehabilitation protocols.

## Introduction

Occupational injuries constitute a substantial global health and economic burden, with the International Labour Organization estimating treatment costs and productivity losses totalling approximately 3.9% of global gross domestic product [1-3]. Upper-limb injuries represent a significant proportion of workplace trauma, frequently requiring surgical intervention and prolonged rehabilitation periods that impact both individual workers and broader healthcare systems.

In developed countries, comprehensive reporting systems, robust workplace safety legislation, and established occupational health services have substantially reduced injury incidence rates [4]. However, data from low- and middle-income countries remain sparse, with most published studies focusing narrowly on hand injuries rather than comprehensive upper-limb trauma patterns [5].

South Africa faces particular challenges in occupational health surveillance, including systematic underreporting, variable healthcare access, and inconsistent enforcement of workplace safety regulations. These factors contribute to an incomplete understanding of the true burden of occupational upper-limb injuries in the region [6-7].

Gqeberha, the largest city in Nelson Mandela Bay, serves as a major industrial centre in the Eastern Cape province of South Africa, hosting diverse manufacturing sectors including automotive assembly, tire production, agro-processing facilities, and extensive service industries [8].

Industrial workers in Gqeberha, like other workers in South Africa, face significant occupational injury risks arising from multiple environmental domains. Physical hazards include noise exposure, machinery vibration, poor lighting, temperature extremes, and inadequate ventilation within manufacturing facilities. Chemical risks stem from welding fumes, paint vapors, dust, and toxic solvents commonly encountered in automotive and agro-processing industries. Mechanical and ergonomic challenges, such as machinery hazards, repetitive strain, manual handling, and awkward postures in assembly operations, further elevate injury potential. Organizational shortcomings, including insufficient personal protective equipment, poor housekeeping, limited safety training, excessive workload, and fatigue from demanding shift patterns, exacerbate these risks. Additionally, infrastructure-related issues, such as inadequate facility maintenance, limited emergency response readiness, and suboptimal workspace design, compound the occupational hazards. Collectively, these factors contribute to a high incidence of work-related injuries, imposing both personal hardship on affected workers and economic strain on the healthcare system. This underscores the urgent need for comprehensive safety interventions [9-10].

According to the Standard Occupational Classification 2000 [11], workers are categorized into nine major groups: Managers and Senior Officials (e.g., Directors and Chief Executives of major organizations), Professional Occupations (e.g., civil engineers, architects, geologists, etc.), Associate Professional and Technical Occupations (e.g., technicians in various fields), Administrative and Secretarial Occupations (e.g., general office assistants and clerks, etc.), Skilled Trades Occupations (e.g., electricians, carpenters, plumbers, welders, butchers, and glass or ceramic makers, etc.), Personal Service Occupations (e.g., security guards, child carers, hairdressers, undertakers and mortuary assistants, etc.), Sales and Customer Service Occupations (e.g., cashiers, sales and retail assistants, etc.), Process, Plant, and Machine Operatives (e.g., assemblers of vehicle and metal goods, machine operators in various plants, etc.), and Elementary Occupations (e.g., cleaners and domestic workers, messengers, porters, and doorkeepers, etc.).

In South Africa, it is a legal requirement that all companies register their employees under the Compensation for Occupational Injuries and Diseases Act (COIDA), commonly referred to as the Workers’ Compensation Act (WCA). This regulation ensures that employees who sustain injuries or contract diseases in the course of their employment are entitled to medical treatment and compensation, typically within the private healthcare sector. The system functions similarly to an insurance scheme, providing financial and medical security to workers while protecting employers from direct liability for such expenses. Compliance with the WCA not only promotes workplace safety and employee well-being but also strengthens the broader occupational health infrastructure across the country [12-13].

Despite this recognized burden, there is a notable paucity of local research describing the epidemiological patterns and clinical outcomes of occupational upper-limb injuries in South Africa. Understanding injury distribution, causal mechanisms, and return-to-work outcomes is essential for developing evidence-based prevention strategies, optimizing clinical management protocols, and guiding occupational health policy development.

This study aimed to review the epidemiology, injury mechanisms, and return-to-work outcomes of occupational upper-limb injuries treated at a private tertiary hospital over a seven-and-a-half-year period.

## Materials and methods

Study design and setting

This retrospective descriptive study was conducted at a single centre, with all patients treated by the principal author. The study period extended from April 1, 2013, to December 31, 2020, encompassing 7.75 years of data collection. Its objectives were: (1) to describe the demographic and occupational distribution of patients with occupational upper-limb injuries; (2) to identify the common mechanisms and anatomical patterns of injury; and (3) to evaluate return-to-work outcomes and associated recovery times.

Study population and selection criteria

All patients presenting with occupational upper-limb injuries during the study period were considered for inclusion, encompassing a broad range of occupational categories to ensure comprehensive representation of the workforce. This allowed for a detailed assessment of injury patterns and risk factors across diverse professional roles, thereby providing valuable insights into the occupational health and safety landscape within the study population. Eligible participants met the following criteria: aged ≥18 years; sustained an upper-limb injury during employment activities; received treatment and rehabilitation under the care of the principal author (orthopaedic surgeon); and provided written informed consent for participation. Exclusion criteria included polytrauma patients, patients with incomplete or missing medical records, and patient refusal to provide consent for data utilisation.

Data collection procedures

Patient medical files and information from the “Employer’s Report of Injury Form” that accompanied every injured employee were systematically reviewed, and data were collected using a standardised data-collection sheet. Variables captured included demographic characteristics (age, gender, and hand dominance); employment factors (occupation type and industry sector classification); and injury characteristics (mechanism of injury, anatomical type, and location). For patients with multiple injuries to the upper limb, the most severe injury was counted; only patients with isolated upper-limb injuries were included. Temporal factors (day of the week and time of injury occurrence) and outcome measures (return-to-work status, time to work resumption, job redeployment, or employment loss) were also recorded. Fractures and dislocations were confirmed radiographically; soft-tissue injuries that were clearly identifiable on clinical examination and/or during surgery were diagnosed accordingly; and rotator cuff tears were verified through ultrasound examination performed by an experienced musculoskeletal sonographer. Return-to-work data were compiled from multiple sources, patient files when follow-up was ongoing, verified with employers in most cases, and obtained directly from participants only when necessary.

Statistical analysis

All data were entered into a secure database and analysed using R statistical software (version 4.2.0) [14]. Descriptive statistics were calculated as frequencies and percentages for categorical variables and as means with ±SD for continuous variables.

Associations between categorical variables were evaluated using Fisher’s exact test due to some expected cell counts below five. Statistical significance was established at p < 0.05. We assessed the dataset for completeness before analysis and found that all variables were fully observed, with no missing data present. Therefore, no imputation or other data-handling procedures were required. The assistance of a professional biostatistician was sought to ensure methodological accuracy and validity of statistical approaches.

Ethical considerations

This study was conducted following approval from the Human Research Ethics Committee (Medical) of the University of the Witwatersrand (Ethics Reference No: M220851). Prior to data collection, permission to conduct the study was granted by the Chief Executive Officer and the Head of Department at Netcare Greenacres Hospital. Written informed consent was obtained from all participants for the use of their medical records in this study. In instances where participants were unable to provide written consent in person, telephonic consent was obtained, recorded, and securely stored.

## Results

Demographic characteristics

**Figure 1:**
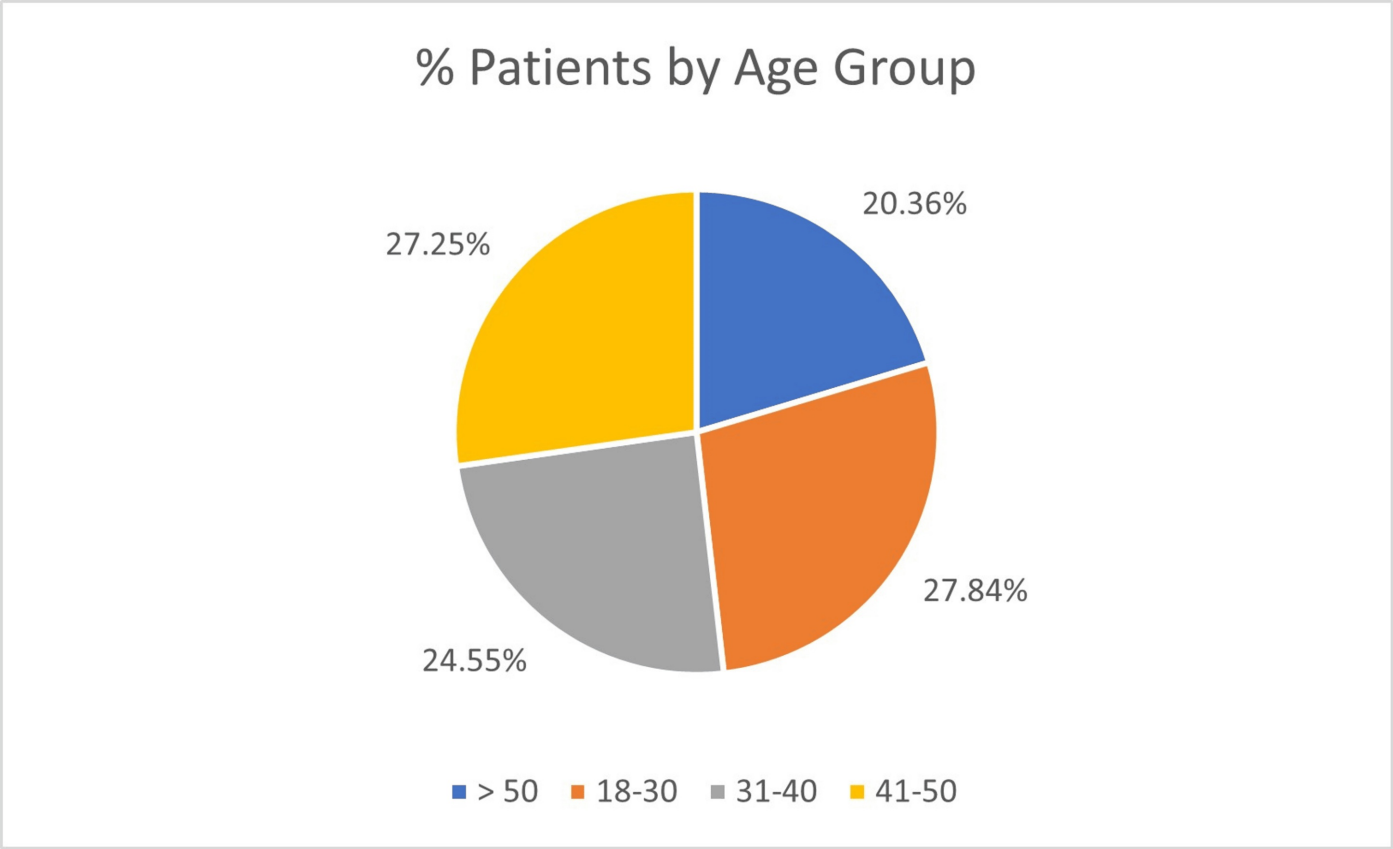
Demographic characteristics of study participants, showing the percentage of patients by age group (N = 334).

**Figure 2:**
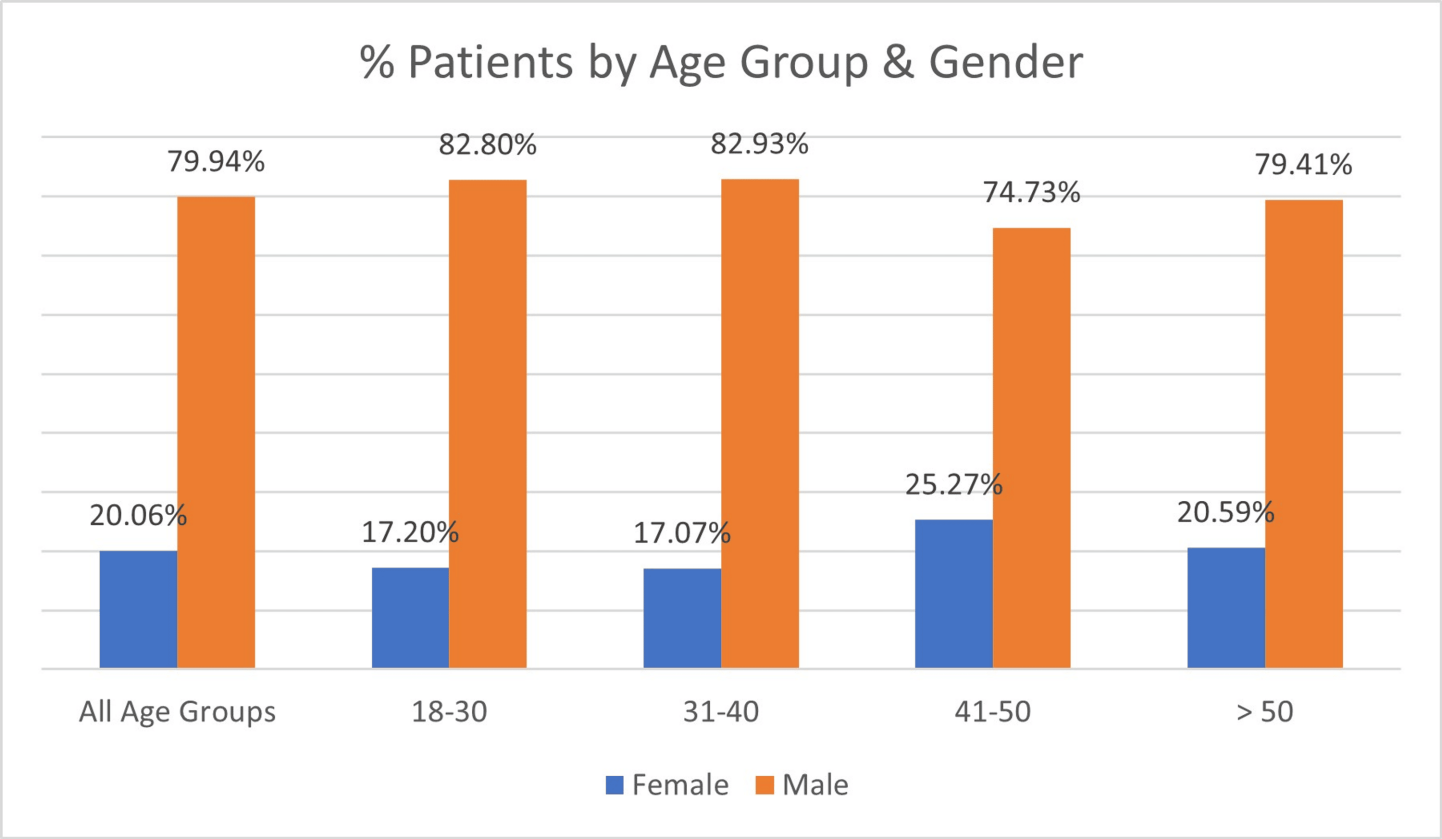
Demographic characteristics of study participants, showing age and gender distribution (N = 334).

**Figure 3:**
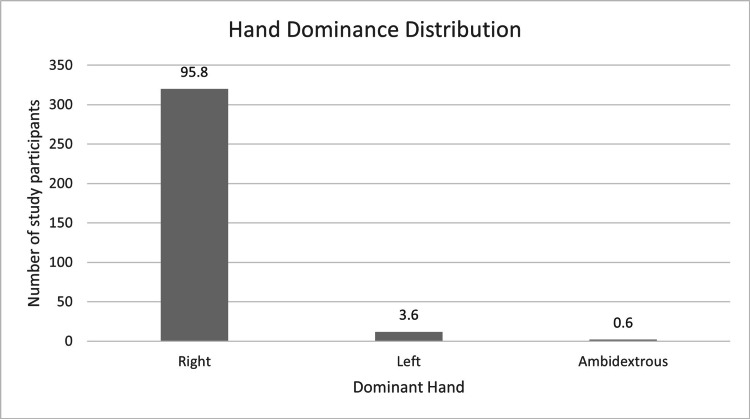
Hand-dominance distribution among study participants, showing 95.8% right-hand dominance (N = 334).

A total of 334 patients were included in the study. Most patients were under 50 years of age (266/334, 79.6%), reflecting the predominance of injuries among workers under 50 years. The age distribution was as follows: 93/334 (27.8%) aged 18-30 years (78 males, 15 females), 82/334 (24.5%) aged 31-40 years (68 males, 14 females), 91/334 (27.2%) aged 41-50 years (68 males, 23 females), and 68/334 (20.3%) aged >50 years (54 males, 14 females), as illustrated in Figures 1-3.

**Figure 4:**
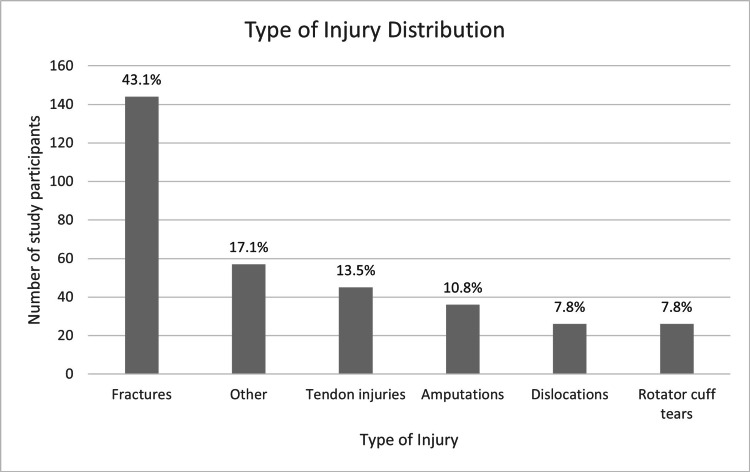
Distribution of injury types among study participants (N = 334).

Fractures were the most frequent injury, accounting for 144/334 (43.1%) of cases, followed by tendon injuries (45/334, 13.5%) and amputations (36/334, 10.8%). Rotator cuff tears (26/334, 7.8%), dislocations (26/334, 7.8%), and other soft-tissue injuries (57/334, 17.1%) comprised the remainder, as illustrated in Figure 4.

The phalanx and distal radius were the most frequently fractured bones, accounting for nearly half of all cases. Less common sites included the clavicle, distal humerus, and scapula. This distribution highlights the predominance of fractures involving the hand and wrist, reflecting the vulnerability of these regions to trauma. Statistical analysis was performed using Fisher’s exact test, with significance set at p < 0.05, as illustrated in Table 1.

**Table 1:** Distribution of fractures by anatomical site among 144 patients. Data are presented as n (%).

Fracture site	n (%)
Phalanx	36 (25.0)
Distal radius	33 (22.9)
Metacarpal	20 (13.9)
Radial head and neck	12 (8.3)
Scaphoid	9 (6.3)
Proximal humerus	9 (6.3)
Radius/ulna shaft	8 (5.6)
Clavicle	5 (3.5)
Distal humerus	5 (3.5)
Olecranon	2 (1.4)
Scapula	2 (1.4)
Coracoid process	2 (1.4)
Humerus shaft	1 (0.7)

The majority of amputations occurred at the distal interphalangeal joint (DIPJ) level, accounting for more than two-thirds of the cases. Fewer patients underwent amputations at the metacarpophalangeal joint (MCPJ) and proximal interphalangeal joint (PIPJ) levels compared with DIPJ, while wrist, below-elbow, and above-elbow amputations were relatively uncommon. These findings indicate that distal-level amputations were substantially more frequent in this cohort than more proximal levels, as illustrated in Table 2.

**Table 2:** Distribution of disarticulation and amputation levels among 36 patients (N=36). Data are presented as n (%). P1: Proximal phalanx; P2: Middle phalanx; P3: Distal phalanx.

Disarticulation/Amputation Level	N (%)
Distal interphalangeal joint (DIPJ) disarticulation / Amputation through P3	26 (72.2%)
Metacarpophalangeal joint (MCPJ) disarticulation / Amputation through P1	5 (13.9%)
Proximal interphalangeal joint (PIPJ) disarticulation / Amputation through P2	2 (5.6%)
Wrist disarticulation	1 (2.8%)
High below-elbow amputation	1 (2.8%)
High above-elbow amputation	1 (2.8%)

Extensor tendon injuries of the digits, involving Kleinert and Verdan Zones I and III, were the most prevalent tendon injuries, representing over half of these cases. Flexor tendon injuries of the digits in Zone II accounted for more than a quarter of the injuries, while biceps tendon injuries were less frequent, with the long head and distal biceps affected in a small proportion of patients. These results highlight the predominance of digital extensor tendon involvement in upper-limb tendon injuries within this cohort, as illustrated in Table 3.

**Table 3:** Distribution of tendon injury types among 45 patients. Data are presented as n (%).

Tendon injury type	n (%)
Extensor tendons (Zones I and III)	27 (60.0)
Flexor tendons (Zone II)	13 (28.9)
Long head of biceps tendon	3 (6.7)
Distal biceps tendon	2 (4.5)

The glenohumeral joint was the most frequently dislocated joint, accounting for over one-third of all dislocations. Elbow and MCPJ dislocations were the next most common, each comprising 15.4% of cases. Acromioclavicular joint dislocations occurred in 11.5% of patients, while lunate, carpometacarpal (CMCJ), and PIPJ dislocations were relatively uncommon. Overall, shoulder dislocations predominated, reflecting their higher susceptibility to traumatic events compared with more distal joints, as illustrated in Table 4.

**Table 4:** Distribution of dislocation sites among 26 patients. Data are presented as n (%).

Dislocation site	n (%)
Glenohumeral joint	10 (38.5)
Elbow joint	4 (15.4)
Metacarpophalangeal joint (MCPJ)	4 (15.4)
Acromioclavicular joint	3 (11.5)
Lunate dislocation (volar)	2 (7.7)
Carpometacarpal joint (CMCJ)	2 (7.7)
Proximal interphalangeal joint (PIPJ)	1 (3.8)

Injuries other than fractures, dislocations, tendon injuries, amputations, disarticulations, or rotator cuff tears were grouped as “other injuries.” Contusions of the hand were the most common injury in this group, accounting for over 40% of cases. Nail-bed injuries and scapholunate ligament injuries were also relatively common, representing 19.2% and 10.5% of cases, respectively. Less frequent injuries included collateral ligament injuries of the thumb, compartment syndrome of the forearm, and foreign bodies in the fingers. Rare but clinically significant findings included nerve-related injuries such as radial nerve injury associated with humeral shaft fracture, long thoracic nerve injury resulting in scapular winging, and compression neuropathy of the median nerve at the hand level, as illustrated in Table 5.

**Table 5:** Distribution of other injuries recorded among 57 patients. Data are presented as n (%).

Type of injury	n (%)
Contusions (hand)	24 (42.1)
Nail-bed injury	11 (19.2)
Scapholunate ligament injury	6 (10.5)
Collateral ligament injury (thumb)	4 (7.0)
Compartment syndrome (forearm)	3 (5.2)
Foreign bodies (fingers)	3 (5.2)
Radial nerve injury associated with humeral shaft fracture	2 (3.5)
Traumatic olecranon bursitis	1 (3.5)
Long thoracic nerve injury (scapular winging)	1 (1.7)
Compression neuropathy of the median nerve (hand level)	1 (1.7)
Foreign body in hand (injected prima paint)	1 (1.7)

**Figure 5:**
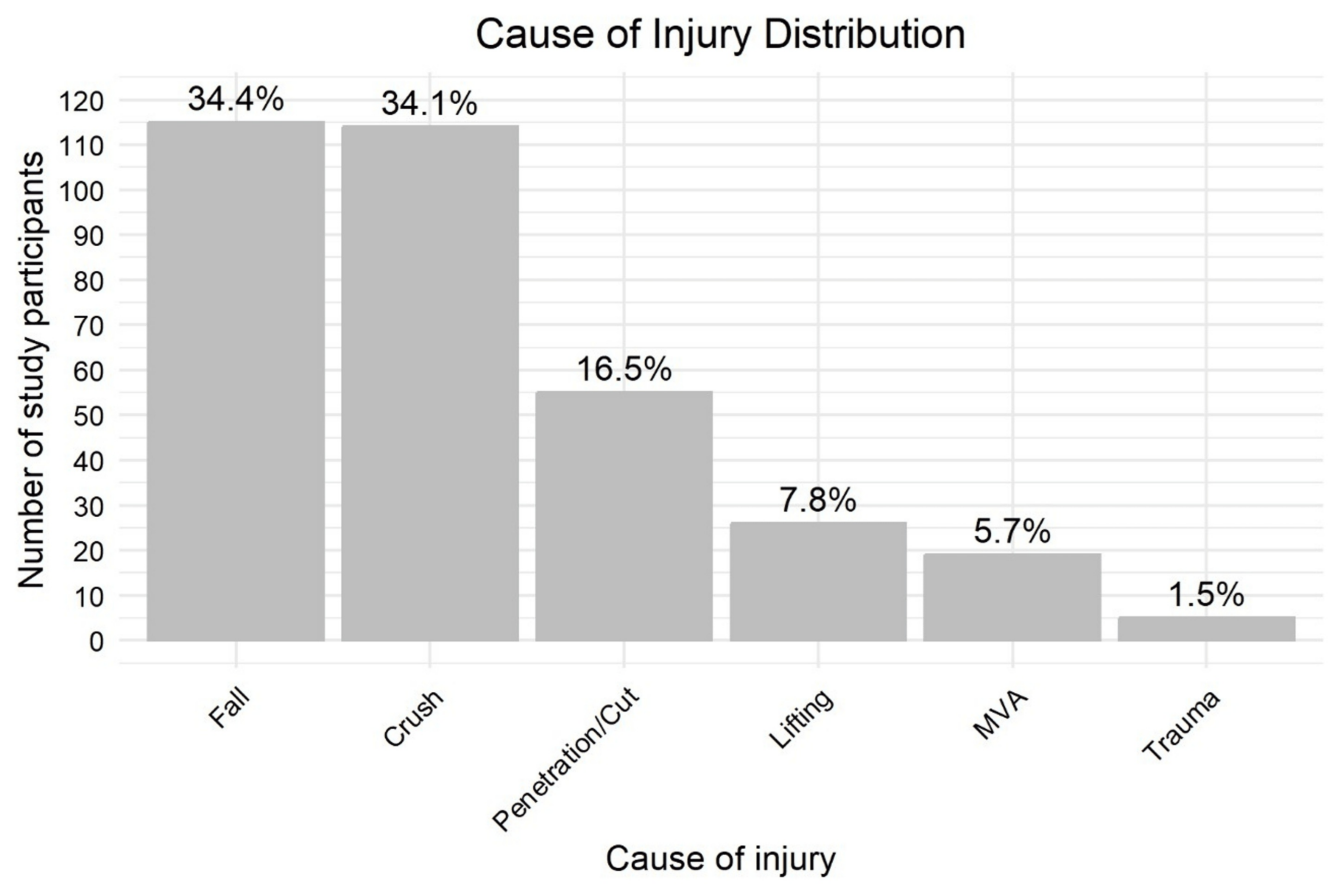
Distribution of injury mechanisms among study participants (N = 334). Data are presented as n (%). MVA: Motor Vehicle Accident.

Falls (115/334, 34.4%) and crush injuries (114/334, 34.1%) together accounted for more than two-thirds of all injuries. Penetrating or cutting injuries accounted for 55/334 (16.5%), while motor vehicle accidents (19/334, 5.7%) and lifting injuries (26/334, 7.8%) were less frequent. Assault-related injuries accounted for a small minority (5/334, 1.5%), as illustrated in Figure 5.

**Figure 6:**
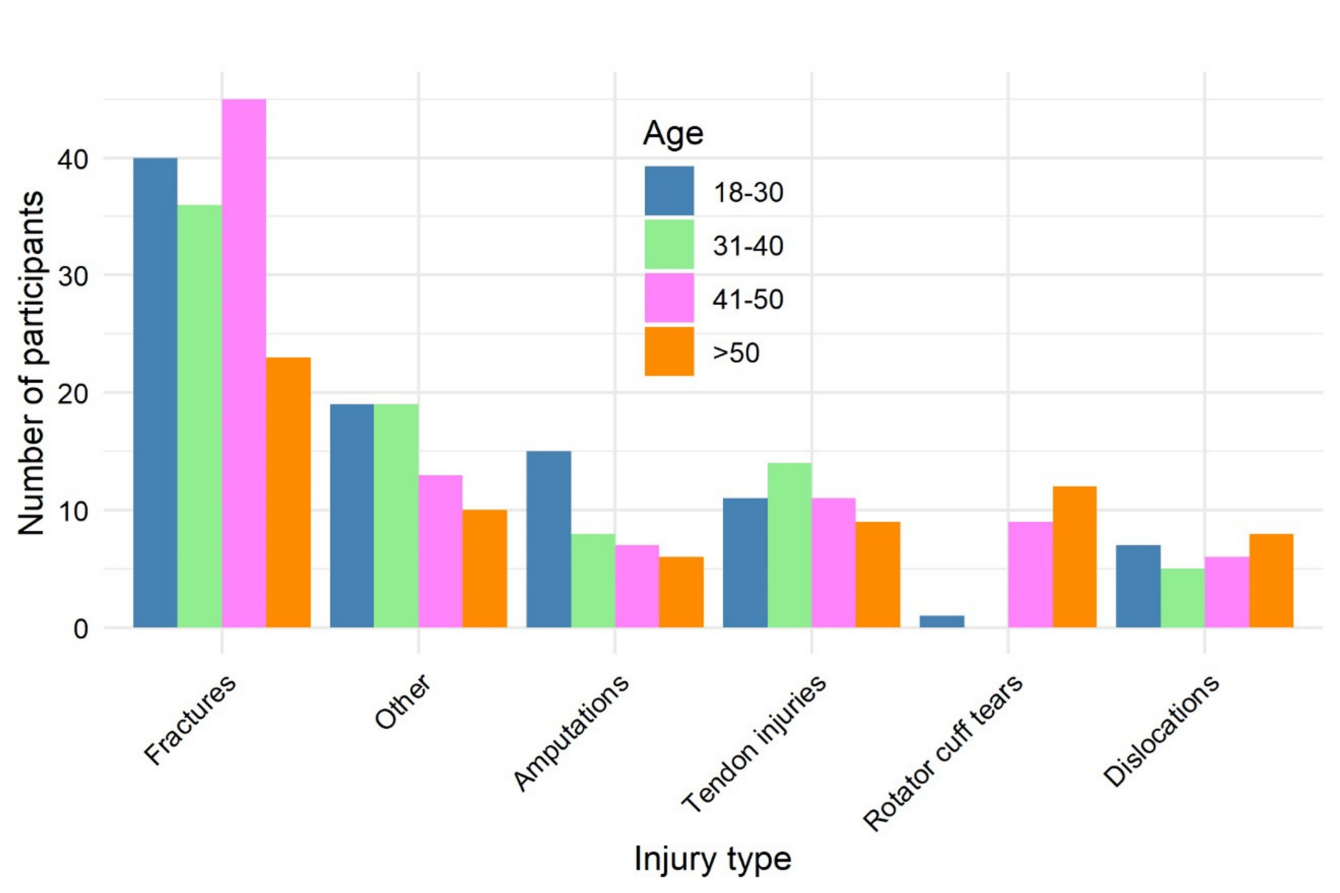
Distribution of injury types across different age groups (N = 334). Data are presented as n (%). Fisher’s exact test: χ² = 45.8; p = 0.0025. Statistical significance set at p < 0.05.

A statistically significant association was found between age group and type of injury using Fisher’s exact test (p = 0.0025). Workers aged 18-30 years were more likely to sustain amputations (18/89, 20.2% vs. 18/245, 7.3% in other age groups), while tendon injuries were most common among those aged 31-40 years (18/98, 18.4%). In the 41-50-year group, fractures were the most frequent injuries (42/88, 47.7%), whereas employees over 50 years of age were more prone to rotator cuff tears (15/59, 25.4%) and dislocations (12/59, 20.3%), as illustrated in Figure 6.

**Figure 7:**
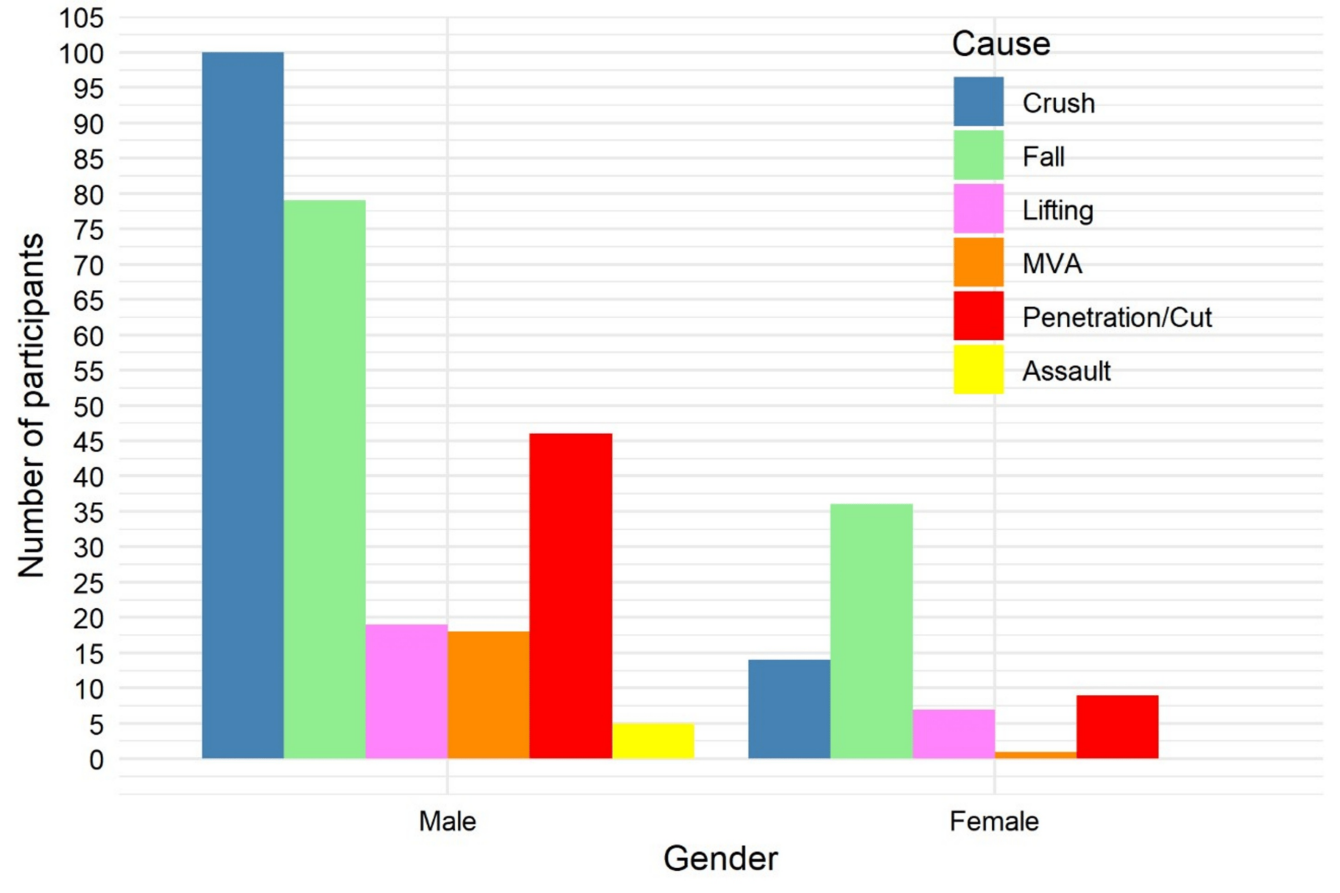
Distribution of injury mechanisms by gender (N = 334). Data are presented as n (%). Fisher’s exact test: χ² = 18.4; p = 0.0035. Statistical significance set at p < 0.05. MVA: Motor Vehicle Accident.

Falls were the leading cause of injuries among females (32/67, 47.8%), whereas falls (83/267, 31.1%) and crushes (98/267, 36.7%) were most common among males. Here, “crushes” refers to any injury in which a portion of the upper limb is caught and compressed between two hard objects. A statistically significant association was observed between gender and cause of injury using Fisher’s exact test (p = 0.0035), as illustrated in Figure 7.

**Figure 8:**
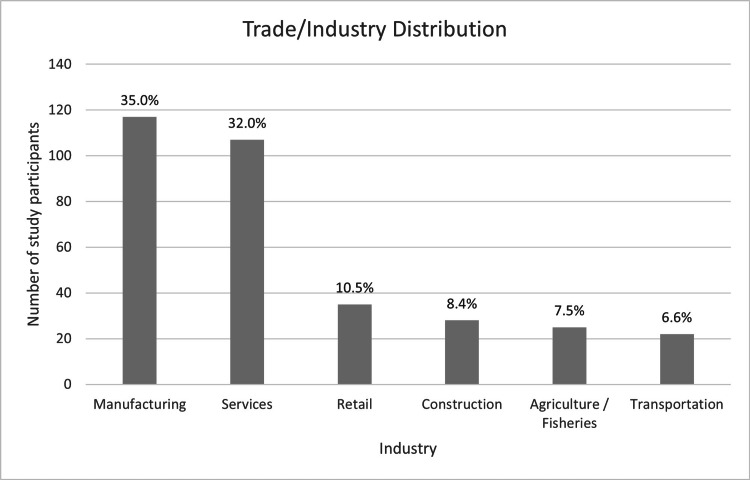
Distribution of injuries across trade and industry sectors (N = 334). Data are presented as n (%).

Most injuries occurred in the manufacturing sector (117/334, 35%), followed by the service industry (107/334, 32%). Smaller proportions came from retail (35/334, 10.5%), construction (28/334, 8.4%), agriculture (25/334, 7.5%), and transportation (22/334, 6.6%), as illustrated in Figure 8.

**Figure 9:**
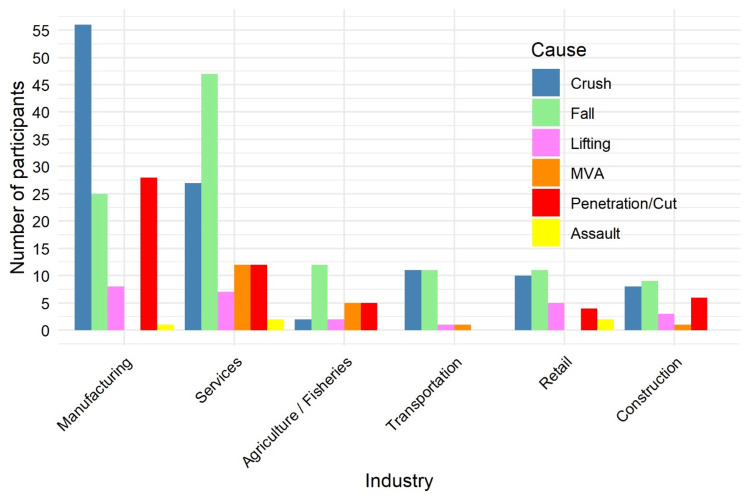
Proportion of injuries by industry sector, showing manufacturing and service sectors as predominant (N = 334). Data are presented as n (%). Fisher’s exact test: χ² = 67.2; p = 0.0001. Statistical significance set at p < 0.05. MVA: Motor Vehicle Accident.

There was a statistically significant association between the cause of injury and the type of industry (p = 0.0005). The manufacturing sector accounted for the highest number of occupational injuries observed in this study. Within the manufacturing industries, crushing emerged as the most frequent mechanism of injury. In contrast, falling was identified as the predominant mechanism of injury across all other industries, as illustrated in Figure 9.

**Figure 10:**
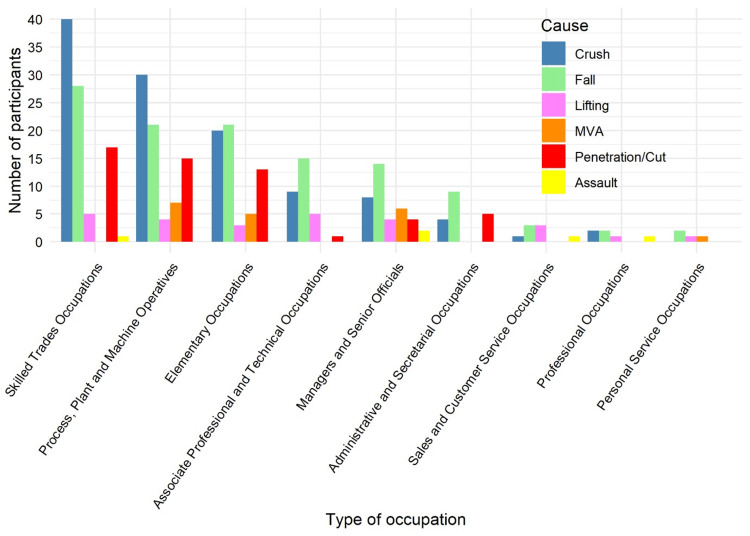
Association between occupation type and injury mechanism (N = 334). Data are presented as n (%). Fisher’s exact test: χ² = 67.2; p = 0.0001. Statistical significance set at p < 0.05. MVA: Motor Vehicle Accident.

A statistically significant association was identified between occupation type and cause of injury using Fisher’s exact test (p = 0.0001). Workers in skilled trades (90/334, 27%), process, plant, and machine operatives (77/334, 23%), and elementary occupations (60/334, 18%) accounted for the majority of reported injuries, which were predominantly caused by crushes, falls, and cutting injuries, as illustrated in Figure 10.

**Figure 11:**
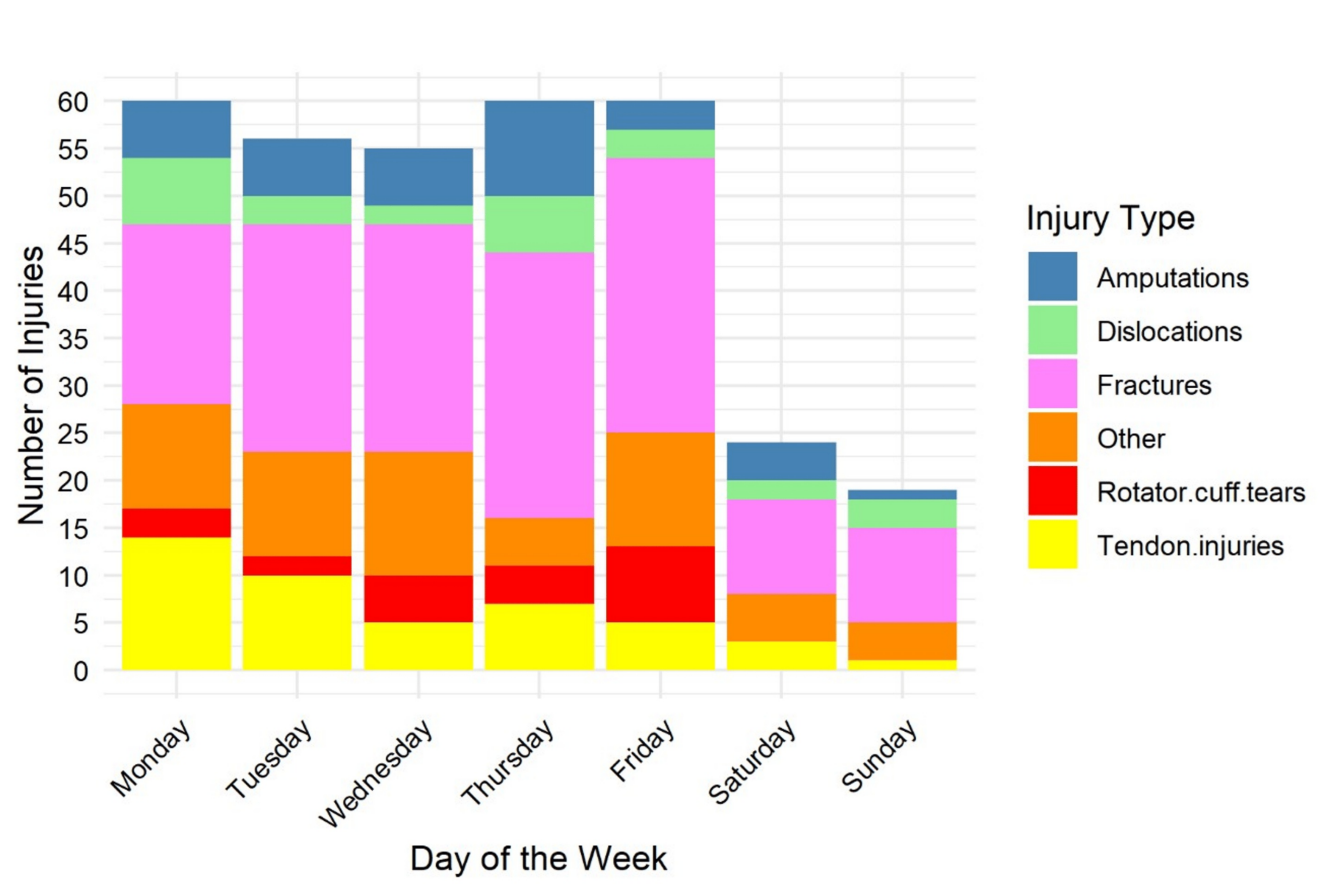
Temporal distribution of injuries by day of the week (N = 334). Data are presented as n (%). Fisher’s exact test: χ² = 12.8; p = 0.0181. Statistical significance set at p < 0.05.

**Figure 12:**
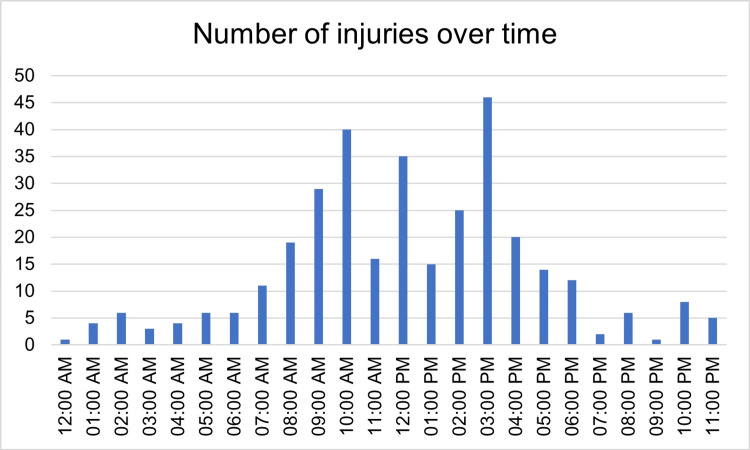
Hourly distribution of injury occurrence, showing peaks at 10:00 AM and 15:00 PM (N = 334). Data are presented as n (%).

Injuries were significantly more frequent on weekdays compared with weekends using Fisher’s exact test (p = 0.0181). Weekday injuries accounted for 278/334 (83.2%) compared with weekend injuries at 56/334 (16.8%). Peaks occurred on Mondays (62/334, 18.6%), Thursdays (58/334, 17.4%), and Fridays (56/334, 16.8%). Injury peaks were most commonly observed at around 10:00 AM (68/334, 20.4%), approximately two hours into the shift, and again at 15:00 (54/334, 16.2%), one to two hours before the end of the working day, as illustrated in Figures 11-12, respectively.

The average time to return to work varied significantly by injury type (ANOVA, F = 3.8, p = 0.003). Rotator cuff tears required the longest mean recovery time, averaging 59.3 ± 28.4 days (range, 28-120 days), while dislocations followed with a mean of 44.2 ± 24.1 days (range, 14-98 days). Amputations and tendon injuries both averaged around 37 days, whereas fractures had a mean recovery period of 35.3 ± 29.7 days (range, 1-120 days). Other soft-tissue injuries showed the shortest recovery time, with an average of 30.8 ± 41.2 days, as illustrated in Table 6.

**Table 6:** Return-to-work time by injury type, showing mean recovery duration. Data are presented as mean ± SD (range, days). ANOVA: F = 3.8; p = 0.003. Statistical significance set at p < 0.05.

Injury type	Mean ± SD (days)	Range (days)	N
Rotator cuff tears	59.3 ± 28.4	28-120	26
Dislocations	44.2 ± 24.1	14-98	26
Amputations	37.1 ± 31.2	14-120	36
Tendon injuries	37.1 ± 38.9	1-155	45
Fractures	35.3 ± 29.7	1-120	144
Other	30.8 ± 41.2	1-180	57

**Figure 13:**
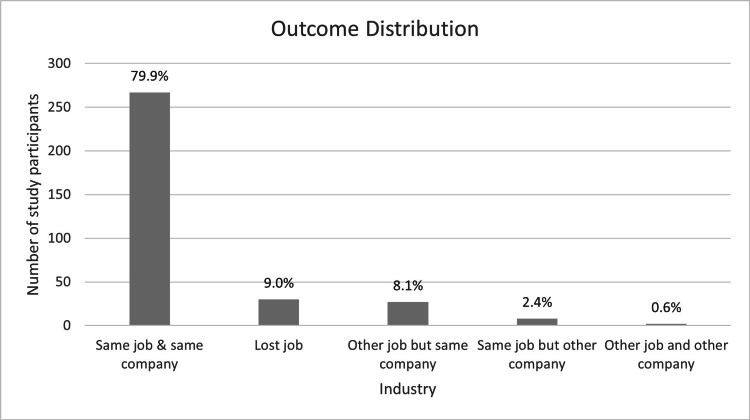
Return-to-work outcomes showing employment status post-injury (N = 334). Data are presented as n (%).

Most employees (267/334, 79.9%) were able to return to the same role with the same employer, while 27/334 (8%) were redeployed to alternative positions. A further 30/334 (9%) lost their jobs, and 10/334 (2.4%) changed employers but remained employed in similar roles, as illustrated in Figure 13.

## Discussion

This study provides one of the largest single-centre reviews of occupational upper-limb injuries in South Africa. The findings demonstrate that these injuries predominantly affect younger male workers in labour-intensive industries, with fractures, tendon injuries, and amputations being the most common. These patterns align with international studies that consistently identify young, active workers as a high-risk group due to greater exposure to machinery, repetitive manual tasks, and higher levels of risk-taking behaviour [13,15-17]. Fractures accounted for almost half of all cases (43.1%), a result consistent with reports from both developing and industrialised settings [13,18]. Phalangeal and distal radius fractures were the most frequent, reflecting the high proportion of crush and fall mechanisms in this cohort. The predominance of extensor tendon injuries in manufacturing workers is notable, as this has not been well described in local literature [13,19].

Amputations mainly affecting digits were most common in workers under 30 years (20.2%), suggesting that inexperience, inadequate training, and reduced supervision may contribute to these devastating injuries [13,16]. Conversely, rotator cuff tears and dislocations were more frequent in workers over 50 years (45.7% combined), probably reflecting occupational strain and injury superimposed on pre-existing degenerative disease [20]. Falls and crush injuries were the leading mechanisms of injury, together accounting for more than two-thirds of all cases (68.5%) [17,21,22]. This distribution is consistent with previous African studies, where inadequate enforcement of safety regulations, absence of machine guarding, and poor adherence to protective equipment contribute to high injury rates [13]. Service-industry workers, particularly women, sustained a higher proportion of fall-related injuries (47.8%), while men in manufacturing were more likely to suffer crush injuries (36.7%), highlighting gendered occupational risks [17,19]. The temporal trends identified, peaks at 10:00 AM and 15:00 PM, as well as higher weekday injury rates (83.2%), are consistent with studies showing increased injuries at times of reduced concentration, fatigue, or work pressure [16]. Encouragingly, most workers (80%) returned to their original roles. However, approximately one in ten (9%) lost employment following their injury, highlighting the substantial socioeconomic burden associated with occupational upper-limb trauma [23]. Return-to-work times were significantly longer for rotator cuff tears (59.3 ± 28.4 days) and dislocations (44.2 ± 24.1 days), particularly among workers over 50 years, reflecting both the physiological demands of these injuries and the slower recovery associated with age [20]. However, we emphasise that the associations reported in this study are correlational, not causal, consistent with the descriptive and retrospective design. The results underscore the need for standard workplace safety protocols, especially in the manufacturing and service industries. Enhanced machine guarding and regular maintenance, combined with structured training programmes tailored for young and inexperienced workers, can significantly reduce workplace injuries [19]. Regular occupational health surveillance is essential, particularly for older employees who may be more vulnerable to degenerative injuries. Careful shift scheduling that considers predictable peak injury times can help minimise risks [16]. A multidisciplinary approach involving occupational therapists, physiotherapists, orthopaedic surgeons, and employers is critical to optimise recovery and reintegration into the workforce.

Limitations

The retrospective, single-centre design of this study limits generalisability, and reliance on private-hospital data excludes public-sector cases, where the injury profile may differ. Under-reporting remains a challenge, as workers without medical insurance or formal employment contracts may not present to the hospital. Future prospective, multicentre studies are essential to validate these findings, assess long-term functional outcomes, and inform the development of a standardised national reporting system for occupational injuries.

## Conclusions

Occupational upper-limb injuries in this study were most common among men under 50 years, particularly in manufacturing and service industries. Fractures were the predominant injury (43.1%), while falls and crush injuries accounted for most cases (68.5%). Younger workers were more prone to amputations (20.2% in the 18-30 age group), whereas older workers sustained more rotator cuff tears and dislocations (45.7% combined in the >50-year age group). Most patients successfully returned to their original roles (80%), but approximately one in ten (9%) lost employment, underscoring the social and economic burden of these injuries. Recovery times varied significantly by injury type, with rotator cuff tears requiring the longest rehabilitation period (59.3 ± 28.4 days).

A coordinated, multidisciplinary approach incorporating early rehabilitation, occupational health support, and strict workplace safety measures is essential to optimise outcomes. Future multicentre, prospective studies are needed to further define injury patterns across sectors, evaluate functional recovery, and inform national occupational health policy in South Africa. These findings emphasise the critical importance of targeted prevention strategies, particularly for vulnerable age groups and high-risk industries, to reduce the substantial human and economic costs associated with occupational upper-limb injuries.
